# Health-Related Rehabilitation after the 2008 Great Wenchuan Earthquake in China: A Ten Year Retrospective Systematic Review

**DOI:** 10.3390/ijerph17072297

**Published:** 2020-03-29

**Authors:** Joseph Kimuli Balikuddembe, Xinglin Zeng, Chuandong Chen

**Affiliations:** 1 Institute for Disaster Management and Reconstruction, Sichuan University and Hong-Kong Polytechnic University, Chengdu 610000, China; leochancd@163.com; 2 Rehabilitation Department, West China School of Medicine, Sichuan University, Chengdu 610000, China; 2016141624073@stu.scu.edu.cn; 3 West China School of Public Health, Sichuan University, Chengdu 610000, China

**Keywords:** health-related rehabilitation, Wenchuan, Sichuan, earthquake, China, systematic review

## Abstract

Being one of the world’s seismically hazard-prone countries, the People’s Republic of China (PRC) witnessed an 8.0-magnitude earthquake on May 12th 2008—which was reported as one of the most destructive disasters since its founding. Following this earthquake, rehabilitation was greatly required for survivors to enable them to achieve and maintain optimal independence; functioning; full physical, mental and social ability; inclusion; and participation in all aspects of life and environments. We conducted a systematic review based on Preferred Reporting Items for Systematic Reviews and Meta-Analyses (PRISMA) guidelines to retrospectively identify, in five English databases/sources, the existing evidence about the Health-Related Rehabilitation (HRR) that was rendered to the survivors of the 2008 Wenchuan earthquake between 2008 and 2018. Only 11 studies out of 828 initial studies retrieved were included in our study and reported the survivors of the 2008 Wenchuan earthquake to have been aged between 10.5 and 55.7, and predominantly diagnosed with posttraumatic stress disorders. Their HRR was mainly premised on physical and physiological therapies, as well as traditional Chinese medicine and digital technologies. Although all HRR interventions used were reported to be effective, none was identified as much more effective than the others in the post-earthquake era —which calls for more robust research to build upon our systematic review.

## 1. Introduction

The People’s Republic of China (PRC) is one of the most seismic hazard-prone countries in the world given its location at the junction of the circum-Pacific seismic belt and the Mediterranean Himalayan seismic belt [1]. As a result, the country has a historical legacy of fatal earthquakes which have stuck with it over the last 100 years [2,3,4]. Almost all types of natural disaster have been reported in China with the exception of volcanic eruptions [1], including—among others—floods, droughts, landslides, mudslides and typhoons [1,4,5,6]. China’s proneness to disasters is attributed to its vast territory, various climatic zones, complex geographical environment and fragile ecological conditions [7]. With respect to earthquakes, the western and eastern parts of PRC are reported to be the most earthquake-prone areas. Accordingly, earthquakes constitute one of the most deadly types of natural disaster, associated with adverse impacts whenever they occur in terms of mass fatalities, injuries, disabilities, morbidity, displacement, posttraumatic stress disorders (PTSD), extensive property damage and devastating economic losses [2,6,8,9,10,11,12,13,14]. On the afternoon of May 12th 2008, the earthquake of 8.0-magnitude (Mw) on the Richter scale, that was reported as one of the most destructive disasters since the founding of PRC, majorly struck Wenchuan country—located in Sichuan Province, a mountainous region in the Southwest of China [3,4,9,10,14,15]. The death toll due to this earthquake was estimated at over 87,000, there were 18,618 missing persons and 374,000 injured, and it nearly left over 45 million people affected in one way another, for example, in terms of being traumatized, displaced and contracting various diseases [4,6,9,10,16,17]. On the other hand, the earthquake’s total economic damage was estimated at over 8,451.0 hundred million Chinese Yuan (¥) [4].

Apart from disintegrating the family and social networks normally depended upon by people [18], disasters such as earthquakes also inhibit economical livelihoods, as well as the delivery of essential needs and services, for instance, healthcare, food, clean water, housing and education. This happens following the damage or destruction of essential infrastructure such as hospitals, buildings and roads, and water, electricity, gas and telecommunication facilities [4,6,8]. More so, earthquakes are known to be associated with high mortality, morbidity and disability rates, which are aggravated by the multiple and complex injuries they cause, for example, traumatic brain injury (TBI), spinal cord injury (SCI), fractures, crush injuries, penetrating wounds or soft wounds [9,11,17,19]. This situation was not exceptional with the 2008 Wenchuan earthquake—which, as mentioned above, contributed to over 374,000 injuries and other impacts [6,9]. Arguably, apart from the emotional and mental consequences, earthquake-induced injuries and disability affect the functional ability, independence and social participation of victims [20]. Dealing with a high number of injured individuals after earthquakes is challenging, and if they are inadequately and not timely identified for comprehensive diagnostics, emergency and formalized treatment [11], many avoidable deaths and life-changing disabilities among survivors can be caused. The challenge, however, is how the various needs of and services for victims related to health, economic, social and cultural aspects can be appropriately rendered and sustained in the long term, especially after the occurrence of large-scale earthquakes—which are known to overwhelm available resources. 

Earthquakes not only increase the degree of vulnerability of individuals but also mean that the those who are injured or disabled, and victims with disabling health conditions, become dependents and become reliant on equipment and supplies, and their immediate families, governments and other providers. In this case, the victims require special attention to meet their essential needs, as well as extramural support to carry out their activities of daily living (ADL) such as walking, eating, bathing, dressing, toileting, transferring and others [21]. With this situation in mind, the role of rehabilitation cannot be emphasized enough to assist individuals who experience or are likely to experience disability after earthquakes, in order to achieve and maintain optimal independence; functioning; full physical, mental, social and vocational ability; and inclusion and participation in all aspects of life and environments [22,23]. Rehabilitation is emphasized not only as an essential component of development but also an inalienable human right to health. So far, this is delineated in the Universal Declaration of Human Rights [24], the 2030 Rehabilitation Agenda [25], the World Health Organization’s Global Disability Action Plan (2014−2021) [26] and the United Nations Convention on the Rights of People with Disabilities (CRPD) [27]. Rehabilitation is usually provided by a mix of family, friends, community volunteers, and professionals or nonprofessional personnel, either in the community or in rehabilitation centers [22,23,25,26,28].

Although it is now over ten years since the 2008 Wenchuan earthquake occurred, its devastating impacts continue to reverberate in the minds of the Sichuanese people, especially in the prefectures or counties of Wenchuan, Mianzhu, Qingchuan, Shifang, Dujiangyan, Beichuan and Qiang Autonomous County—where the majority of the causalities were witnessed [4,16,20]. Immediately after the 2008 Wenchuan earthquake struck and to date, the central and provincial governments of PRC and other stakeholders rapidly responded to the disaster by embarking on several post-earthquake interventions, including rehabilitation care, as reported in a substantive body of literature [1,3,4,5,9,10,19]. Ideally, planning for rehabilitation services for populations in the aftermath of earthquakes and others disasters is paramount to help to support not only the survivors with long-term disabilities and those living with different disabling conditions, but also those involved in the relief process, including the health-care workers [10]. Moreover, in the quest of achieving the Sendai Framework for Disaster Risk Reduction 2015−2030—a global strategy for addressing disaster risk and resilience—the need for rehabilitation is recognized throughout [29]. Therefore, through a systematic review, the present study aimed at retrospectively identifying the existing evidence about the health-related rehabilitation that has been rendered to the victims or survivors of the 2008 Wenchuan earthquake between 2008 and 2018. Ultimately, the findings of this study are intended to help to inform further interventions for effective rehabilitation services, particularly to the survivors of earthquakes with life-long disabling conditions, not limited only to those of the 2008 Wenchuan earthquake but also those of other earthquakes and disasters elsewhere.

## 2. Materials and Methods 

### 2.1. Study Design

This review was conducted by following the Preferred Reporting Items for Systematic Reviews and Meta-Analyses (PRISMA) guidelines [30], to identify the existing evidence about the health-related rehabilitation (HRR) interventions which have been rendered to the survivors or victims of the 2008 Wenchuan earthquake between 2008 and 2018. We defined HRR based on Article 26 (Habilitation and Rehabilitation) of CRPD which outlines the measures States Parties should undertake to ensure that PWDs can access health-related rehabilitation... *[Sic]...*including through peer support, to enable them to attain and maintain their maximum independence; full physical, mental, social and vocational abilities; and full inclusion and participation in all aspects of life [23]. HRR is diverse in terms of target population interventions (rehabilitation medicine, orthopaedic surgery, physiotherapy, speech and language therapy, occupational therapy and assistive devices) and outcomes.

### 2.2. Inclusion and Exclusion Criteria

The inclusion criteria of our systematic review focused on retrospectively identifying the studies of interest that reported the following: 1) injured survivors or victims (as the target population) who were negatively impacted in terms of physical, mental or psychological functioning, activity and participation limitation following the 2008 Wenchuan or 5.12 earthquake; 2) primary research studies (with original designs) that reported different HRR interventions or programs, for example, those based on physical, occupational and psychological therapies, traditional Chinese Medicine, education and non-Pharmaceutical therapy; 3) studies conducted within China, and with particular focus on the 2008 Wenchuan earthquake; and 4) studies published in English within the period between 2008 and 2018. We excluded studies based on the following: 1) with a scope and main focus not related to the 2008 Wenchuan earthquake; 2) without an original study design; 3) published before May 12th 2008 and after 2018; 4) not published in English; and 5) literature reviews, news pieces, editorials, narrative descriptions, opinions, commentaries, health policy or emergency response studies, pharmaceutical studies, surgical or rehabilitation studies, case reports, commentaries, letters to the editor, and conference proceedings.

### 2.3. The Search Strategy

Initially, based on the abovementioned inclusion criteria, three independent reviewers (JBK, ZX and LC) extracted the relevant studies or articles in five English databases/sources including PubMed, Science Direct, Springer Link, Web of Science and Google Scholar. The period between May 12th 2008 and after May 12th 2018 was considered since it marked the tenth (10th) anniversary of the 2008 Wenchuan earthquake. Following the PRISMA guidelines, the search strategy involved using a mix of MeSH keywords and free text terms as follows: ((((injuries) OR pain)) AND (((((((rehabilitation) OR physical therapy) OR occupational therapy) OR psychological therapy) OR education) OR non-Pharmaceutical therapy) OR traditional Chinese medicine)) AND (((Wenchuan earthquake) OR 5·12 earthquakes) OR Sichuan earthquake).

### 2.4. The Data Abstraction Process

At first, the search results were critically read based on their titles, abstracts and keywords. Their related information was abstracted using Microsoft Excel as follows: the title, the publication date of the study, the setting where the study was conducted, research questions/hypotheses, the study design, the population subtype/participants, the sample size, the intervention and the key findings or recommendations. This helped with the quick identification and screening of eligible studies meeting our study criteria. Afterwards, the full texts of studies identified eligible were retrieved and stored in an Excel Spreadsheet and in the EndNote software X8 (Thomson Reuters, Philadelphia, United States). At this stage, we identified and eliminated any duplicates found. The details of retrieved studies were categorized according to the five respective databases/sources from which they were retrieved, as well as the types of their designs, which mainly included cross-sectional, clinical, randomized controlled trial (RCT), case-control, data analysis and longitudinal designs. Additionally, the references of eligible studies were further explored for the purpose of identifying more potential and relevant studies that would have been missed in the initial search results. 

### 2.5. Screening and Study Selection

Two reviewers (JBK and ZX) critically read and screened the searched studies based on their titles, abstracts and keywords before retrieving and storing their retrieved full texts in both a Microsoft Excel Spreadsheet and in the EndNote software X8, as noted above. Using the Population Intervention Comparison Outcome (PICO) method [31,32], details of the eligible studies were sub-grouped according to the following: the cities/hospital, the study design, interventions, the study purpose, the subject age/gender, the numbers of patients, the study type, primary outcomes and recommendations/primary findings of the study. Each article (study) was still reviewed independently by JBK and ZX, and in the case of any discrepancies emerging regarding their eligibility, the two reviewers resolved them by discussion. Regarding this, no case necessitated the intervention of a third reviewer. During this stage, the reliability and validity of each of the studies reviewed was ensured. At the end of the screening process, studies eligible for final inclusion in our systematic review were identified and also categorized either as RCT or non-RCT.

### 2.6. Quality Assessment

An analysis of the methodological quality was done by JBK and ZX to assess the risk of bias for all RCT studies retrieved using the Cochrane Collaboration’s risk of bias tool [33]. This was aimed at assessing their risk of bias, rigor, and transparency. The Cochrane Collaboration’s risk of bias tool contains seven items, namely random sequence generation (selection bias), allocation concealment (selection bias), the blinding of participants and personnel (performance bias), the blinding of outcome assessment (detection bias), incomplete outcome data (attrition bias), selective reporting (reporting bias) and other bias. All included studies were rated as having a low, unclear or high bias based on the seven items. Besides, we used the Risk of Bias in the Non-randomized Studies of Interventions (ROBINS-I tool, innovated at University of Bristol, Bristol, United Kingdom) [34] to assess the non-RCT studies for their quality. ROBINS-I is similarly based on the Cochrane Risk of Bias tool for RCTs and consists of seven domains: confounding, selection, departures from intended interventions, missing data, measurement of intervention, outcome measurement and selective reporting [33].

## 3. Results

### 3.1. Search Results

**Figure 1:**
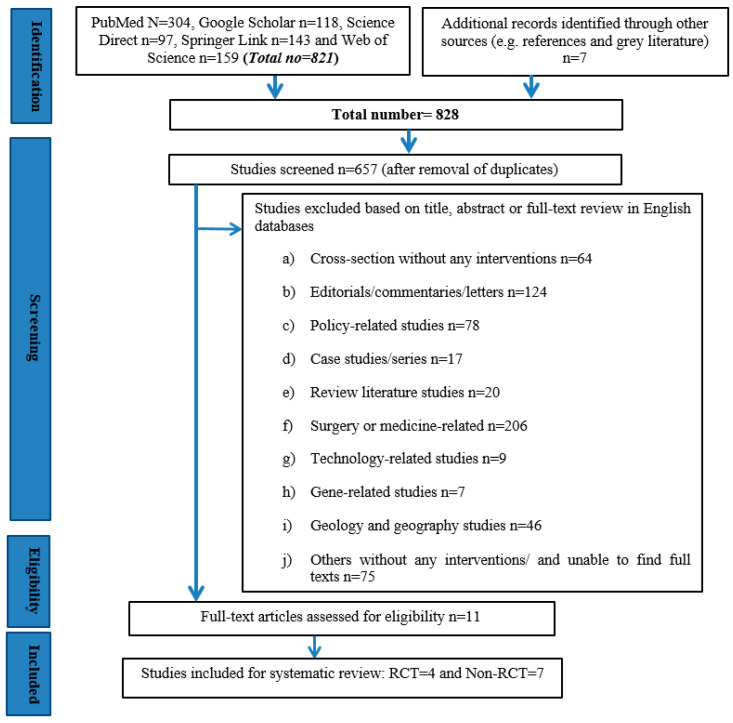
A flow chart indicating the processes that were involved in identifying, retrieving and screening the eligible studies for the systematic review, based on PRISMA.

Initially, a total of 821 studies were electronically retrieved from the five English databases (Figure 1). An additional seven potentially relevant studies were also identified through searching the references of the 821 studies. As a result, this yielded a total of 828 records as eligible studies for screening in our systematic review. After conducting all of the relevant screening, including removing the duplicates, the full texts of only 11 studies finally met the inclusion criteria in line with the purpose of our systematic review. A summary of the characteristics of the 11 studies [16,17,19,20,35,36,37,38,39,40,41] is presented in Table 1. The 11 studies were published between 2012 and 2019, the majority of them in 2013 [16,17,36] and 2014 [37,38,39], whereby three studies (n = 3) were published in each year. In this case, no study was retrieved that had been published in the immediate aftermath of the 2008 Wenchuan earthquake.

### 3.2. Characteristics of Participants

The identified studies predominantly investigated the victims who were diagnosed with PTSD (*n = 6*) [35,36,37,38,39,41], followed by limb amputees (*n = 2*) [20,40]. In other studies, the participants included those with SCI (*n = 1*) [19], fractures (*n = 1*) [17] and general disabling injuries (*n = 1*) [16]. Although all of the 11 studies enrolled their samples of participants from the survivors of the 2008 Wenchuan earthquake, only seven of them (n = 7) [16,17,19,20,35,36,39] reported the specific locations where they were recruited from, and particularly included the most severely affected counties or cities of Beichuan, Mianzhu, Dujiangyan and Jiangyo. Other participants were recruited from outside of the earthquake zone and mainly from the hospitals where they were receiving various kinds of treatment, such as West China Hospital - Chengdu, Sichuan Provincial People’s Hospital, the Shifang Counseling Center and Shifang People’s Hospital. All of these hospitals are located in Sichuan province, southwest of PRC.

### 3.3. Mean Age and Gender

The mean age of participants in each study ranged from 10.5 [37] to 55.7 [36] years. Apart from the two studies (*n = 2*) which directly dwelt on children, adolescents or youths [37,38], seven studies (*n = 7*) reported the age range of participants to be, on average, between 16 and 85 years [17,19,20,35,36,40,41], while other studies did not report any age ranges. All of the 11 studies involved the participants of both genders (sexes), whereby 63.4% of all of the total participants were females, and the highest number of them was reported to be 296 [17].

### 3.4. Study Designs

A combination of qualitative and quantitative methodologies based on the cross-sectional approach was used in the 11 studies and included case-control, retrospective and prospective cohort, questionnaire or interview survey, and longitudinal quasi-experimental designs. Data were directly obtained from the populations (subjects) investigated using different data collection tools. Of 11 studies, a randomized control trial (RCT) was employed in four studies (*n = 4*) [35,36,38,39], where different participants were randomly allocated to either intervention or non-intervention groups, in order to receive different HRR interventions. Almost all of the studies were designed and championed by academic-based institutions and mainly by the universities, although with financial support from the various funding stakeholders like the Chinese government, charities and foundations, as well as the support from volunteers like hospital staff for data collection.

### 3.5. Interventions and Settings

Apart from the three studies (*n = 3*) where regular physical rehabilitation was used, other studies were blended with a different mode of rehabilitation interventions which included narrative Exposure Therapy (NET), cognitive-behavioral therapy (CBT), Xiao-Tan-Jie-Yu-Fang (XTJYF)—a Chinese herbal formula, Interpersonal Psychotherapy (IPT), NHV rehabilitation programming, Stand Tall rehabilitation, calligraphic training and Web-based self-help rehabilitation programs. While the Stand Tall rehabilitation intervention was jointly delivered at the clinic, school and home [40], the rest of interventions were exclusively delivered in the hospitals [16,19,20,40] or community-based settings [17,35,36,39,41] and followed by the schools [37,38]. The delivery of some interventions like CBT [38] was group-based, on the other hand. The shortest and longest delivery times for some of HRR interventions identified in the studies were 30 days and 5 years, for calligraphic training [37] and institution-based rehabilitation [20], respectively. In this case, there was no particular timeframe specified or recommended for the identified interventions.

### 3.6. HRR Outcomes

Analysis of the 11 studies showed that many of them (*n = 6*) intended to assess PTSD or major depressive disorder (MDD) [35,36,37,38,39,41] as the primary outcome(s) for HRR, while others explored the physical functioning and QoL of participants. Among the most prevalent symptoms to have aroused PTSD or MDD, as revealed in six studies, were anxiety and depression, general mental stress, anger and interpersonal violence, and other mental disorders. Accordingly, there was both a variation in and a combination of the different tools used to measure these outcomes. They included the Impact of Event Scale-Revised (IES-R) [36], the Children’s Revised Impact of Event Scale (CRIES-13) [37,38], the Center for Epidemiologic Studies Depression Scale (CES-D), the Connor-Davidson Resilience Scale (CD-RISC) [38], the Symptom Checklist-90-Revised (SCL-90-R) [35], the Clinician-Administered PTSD Scale (CAPS), the PTSD diagnosis and Structured Clinical Interview for DSM-IV (SCID) for MDD diagnosis [39], and the PTSD Checklist-Civilian Version (PCL-C) [17]. Others were the Barthel Index (BI) [16,19,20], the Visual Analogue Scale (VAS), the Medical Outcomes Study Short-Form 36 (SF-36) [20], the Amputee mobility predictor (AMP) (AMPPro version) [40], the Checklist 90-Depression (SCL-D), the Trauma Coping Self-Efficacy scale (CSE), the Crisis Support Scale (CSS) and the Social Functioning Impairment questionnaire (SFI) [41]. Based on these outcome measurement tools, CRIES-13 and BI were applied in more than two studies. 

### 3.7. Quality Assessment and the Risk of Bias

**Figure 2:**
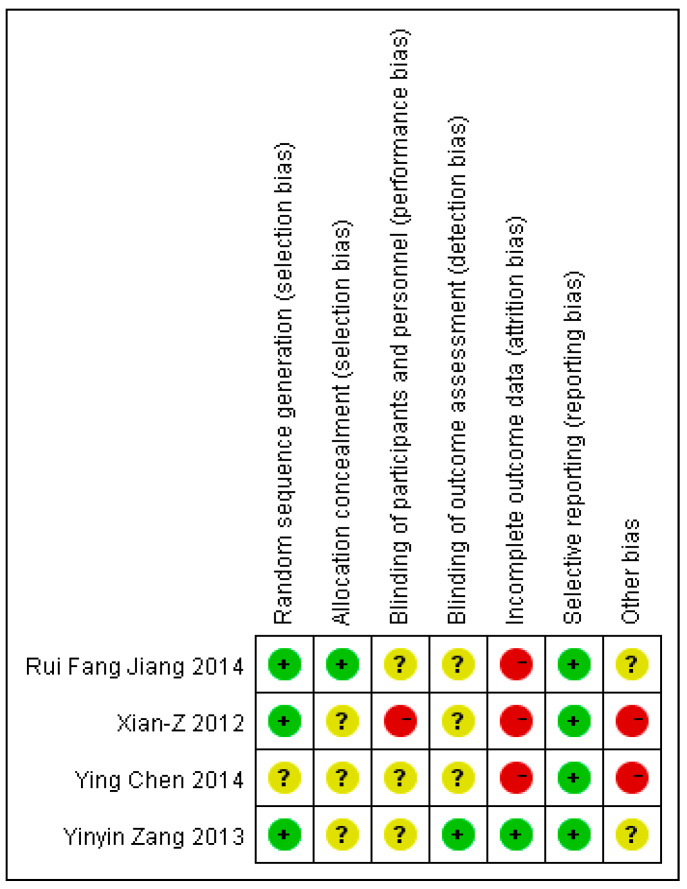
Quality assessment of the randomized controlled trial (RCT) studies that were included in the review based on the Cochrane Collaboration’s risk of bias tool.

Figure 2 and Table 2 summarize the different aspects concerning the methodological quality of the studies based on the Cochrane Risk of Bias Assessment Tool [33] and ROBINS-I [34]. Among the RCT studies (Figure 2), most of the methodological aspects were not well-covered. Four (n = 4) out of seven aspects of the Cochrane Collaboration’s risk of bias tool were covered in one study [36]. ‘Selective reporting’ was the only aspect covered by all four of the RCT studies. On the side of non-randomized studies (Table 2), the domains of ROBINS-I for assessing different types of bias were considered in at least five studies (n = 5); and their overall bias rating was ‘moderate’ [16,17,19,37,41]. The overall biases for the other two studies were rated as critical (n = 1) [20] and serious (n = 1) [40].

## 4. Discussion

This retrospective systematic review provides an insight into the HRR programs that have been rendered to the survivors of the 2008 Wenchuan earthquake over the past ten years, based on 11 studies. Its findings, however, indicate that there is still a paucity of research with robust methodological designs on HRR. This aside, the study highlights that large-scale earthquakes, like the 2008 Wenchuan earthquake, impact and expose the survivors of different ages—from adults to children—to a wide range of health conditions, not limited only to the risks of long-lasting or permanent disabilities emanating from injuries like SCI, fractures, TBI and peripheral nerve injuries [16,17,19,20,40]. In this case, rehabilitation—which is also a critical component of healthcare systems delineating different strategies for health care, prevention, cure, trauma management and support [26,28,42]—is vital in responding to the immediate and long-term health needs of causalities after an earthquake. Rehabilitation is particularly vital for addressing PTSD—which the majority of the included studies identified in our systematic review show to have affected many survivors. PTSD is a psychological disorder caused by unusual threats or catastrophic events such as earthquakes [12,14,15,43]. It can also occur in people who have experienced, witnessed or known about traumatic events that posed to them a risk of death, serious harm or threat to physical integrity, and that as a response to such have felt intense fear, helplessness or horror [44]. Thus, various rehabilitation interventions were warranted in the prevention of the loss of function, the restoration of function and the increase or maintenance of current function [22,23,45], not only for the over 374,000 individuals injured in the 2008 Wenchuan earthquake [6,9], but also for PTSD victims. Rehabilitation is essential for the psychological status of earthquake survivors by improving their wellness, QoL and their performance of ADL, as well as for promoting their social inclusion and participation in community life [22,26]. Also, rehabilitation in disasters and emergencies can help to decrease morbidity and mortality, for instance, that related to suicide attempts, and reduce the length of hospital stays [46,47]. Despite the 11 studies included in our systematic review being heterogeneous—in terms of objectives, target populations, settings, outcomes, methodological designs and durations for implementation—they all reaffirmed an overarching role of rehabilitation in the post-earthquake scenario. 

Given the large-scale nature of the 2008 Wenchuan earthquake, there was indeed a great need for several rehabilitation interventions to be enacted, considering the diversity of the characteristics of the survivors. This can be explained by the 11 studies, which reported participants of varying HRR needs and outcomes such as functioning, mobility, QoL and psychology [16,19,36,37,38,39,40,41] —all of which required either immediate, intensive or long-term rehabilitation of a given duration and at a given location. To a large extent, the interventions reported in 11 studies are heterogeneous and they aimed at addressing the physical, behavioral, psychological, self-management and educational HRR needs of the survivors of the 2008 Wenchuan earthquake. Apart from physical rehabilitation or therapeutic programs, which were dominant, other interventions including NET, CBT, IPT, Stand Tall rehabilitation and calligraphic training were also implemented and reported to be effective in helping the survivors of the 2008 Wenchuan earthquake, especially in improving their functional and QoL outcomes, as well as their abilities to perform ADLs more independently [16,17,19,20,40]. The interventions were also effective in treating and reducing the survivors’ PTSD symptoms like anxiety and depression, and also in enhancing their psychological resilience [35,36,38,39]. These findings are consistent with previous results from a broad systematic literature review and meta-analysis studies that similarly explored the different rehabilitation interventions, services and outcomes in post-earthquake settings, and those following other disasters within and outside China [2,13,14,15,43,44,48,49,50,51,52]. Encouragingly, some of the HRR programs after the 2008 Wenchuan earthquake, such as physical and physiological rehabilitation, commenced within the first 5 months after the earthquake, which is commendable and helped to ameliorate long-term impacts such as injuries and PTSD from turning into acute problems for survivors that would prevent them from returning to normal life.

Ideally, any rehabilitation interventions or programs in situations following earthquakes and other disaster situations must be multidimensional—taking into account the different models of treatment that identify not only the health and physical needs, but also lead to better outcomes for patients. Fortunately, apart from the commonly known rehabilitation interventions, especially the physical and physiological therapies, our systematic review discovered other unique rehabilitation approaches to have been used in rehabilitating the survivors of the 2008 Wenchuan earthquake. They were premised on traditional or indigenous treatments and digital technologies, such as traditional Chinese medicine —XTJYF [35] and web-based interventions [41]. In this case, embracing the traditional rehabilitation treatments was not only helpful in terms of cheaper fees, easy accessibility and enabling the provision of quick services, especially in the resource-constrained settings, but also helped treatment to be embraced in settings like Sichuan province, where traditional medicine is still cherished for good health and treating various symptoms of patients [52,53,54]. Moreover, similar to XTJYF traditional Chinese medicine, for example, ‘Kampo’, a Japanese medicine is reported from ancient times to have been used to treat various infections and traumas, injuries, fractures and pain [52]. On the other hand, harnessing the evolving digital technologies, for example, different web-based applications, can be a pathway for delivering a full spectrum of health services, including self-rehabilitation [28,55] without the need for face-to-face encounters between a patient and a rehabilitation service provider in the post-earthquake scenario. Of note, based on our findings, no specific locations were designated or recommended in the 11 studies for the delivery of the above interventions. Rather, different interventions or programs were rendered to victims at the clinic, or in schools, homes, hospitals or community-based settings. What matters, in this regard, is the identification of a convenient and suitable place that is preferred by the patient; guaranteeing his/her confidentiality, privacy and safety; and above all, not compromising the recommended healthcare standards [23,25,28].

Earthquakes, like any other disasters, are traumatic events and they result in a range of physical, mental and psychological health consequences [2,8,12,13,14,15,43,50,51,52,56]. Indeed, this is attested to in the results of six studies in our systematic review [35,36,37,38,39,41]—which showed a high prevalence of PTSD among the different groups of survivors of the 2008 Wenchuan earthquake. PTSD aroused several symptoms such as anxiety, depression, anger, stress, psychoticism, somatization, sleep disorders, interpersonal sensitivity and violence, as well as thoughts, feelings, or stimuli associated with the traumatic events, among victims [2,12,13,14,15,35,36,37,38,39,41,44,51,52,56,57]. Again, this argument concurs with the findings of an earlier systematic review and meta-analysis studies, which affirmed how a real burden of PTSD is prevalent in many earthquake survivors [12,13,14,15,43,44,49,56,58]. The studies described PTSD as one the most frequent and debilitating psychological impacts associated with high magnitude earthquakes, in addition to other public health challenges like fatalities, injuries, disabilities and disease outbreaks [2,5,8,11]. This reinforces the importance of prioritizing PTSD among the targets of the physical and mental health needs in the post-earthquake scenario to support not only the survivors, but also other persons who are involved in the relief process, including the health-care workers themselves [10]. Understanding the symptoms and patterns of PTSD or MDD in detail may help to find better prevention strategies, develop methods of rapidly assessing the needs of affected and traumatized people and elucidate how to provide their necessary rehabilitation. In one way or another, this can be possible with the help of using various rehabilitation assessment tools like those identified in some studies included in our systematic review, for instance, PCL-C [17], CAPS and MDD diagnosis [39], and SCL-D and CSE [41]. These tools are fundamental in detecting, diagnosing and treating the early symptoms of PTSD before they become acute or chronic among the victims of earthquakes and other disasters. However, the use of such tools should comply with the WHO ICD-10 Classification [56] and also take into account the precarious and widespread economic, social and cultural impacts of earthquakes [2,6,8,9,10,11], if they are to effectively address the PTSD-related reactions of victims, especially in the early post-earthquake period. 

Another pertinent consideration for HRR in the post-earthquake scenario is the need to promote and strengthen the interventions that target specific groups of people within the general community affected—which may be more vulnerable and at greater risk of negative outcomes than others [50]. The most vulnerable groups in disasters and emergencies are identified to include physically or mentally disabled persons (blind, with cognitive disorders or with mobility limitations), elderly persons, women, children, adolescents, ethnic minorities, prisoners, homeless persons, illiterate persons and the economically impoverished [2,10,14,28,50,51]. These groups corroborate with examples that our systematic review identified in some of the included studies, such as the females who were exposed to a higher risk of PTSD [17], illiterate amputees [20], childhood survivors, those with hyperarousal symptoms [37] and the adolescents who lost their parents [38]. It is worth noting that the proportions of elderly (over 65 years) and younger (< 15 years) patients are reported to have been 18.2% and 11.2% of the total, respectively, after the 2008 Wenchuan Earthquake [4]. It is quite clear that earthquakes and other mega disasters overwhelm available resources like health-care, medicine, personnel and infrastructure, as well as rehabilitation services, especially in the less-resourced settings [28,45,46,47]. This can lead to the disregarding of the special needs of some vulnerable groups, like mobility and hearing aids, daily medications and special or preferred food diets, especially in the response phase. As a result, this can aggravate health-related effects and other risks among the victims, such as PTSD and MDD, isolation and loneliness, drug or alcohol abuse, loss of dignity and sense of guilt, doubt, distress and intense fear, vertigo, insomnia, psychoses or even suicidal ideation [15,50]. Besides, negative outcomes from some rehabilitation interventions can be achieved if the needs of vulnerable groups are not adequately addressed. Evidence in this regard is revealed in one study in our systematic review that found no change in the QoL and life satisfaction among the illiterate survivors with amputation [20]. From the HRR standpoint, the needs for the most vulnerable groups during and in the aftermath of earthquakes must be quickly identified by different service providers while rendering rehabilitation care. This helps not only to enhance their functional, and psychological self-care, wellbeing, QoL and adaptive capacity, but also their sense of social belongingness and connectivity, being loved and sympathy from others. Moreover, the level of vulnerability of some groups at greater risk may not only predict and determine the usage of, adherence to and coping with, but also the success of implementing certain rehabilitation programs.

By and large, due to the diversity of health needs and disabling conditions associated with earthquakes, identifying effective rehabilitation interventions or programs and their implementation becomes a challenge at times. One possible solution is to deliver some interventions in groups using RCTs, through which people are allocated ‘at random’ to receive one of several interventions [59]. An RCT is required to detect uncertainty and small-to-moderate, but clinically meaningful, treatment effects between competing interventions. It is of little wonder that the rehabilitation programs implemented through RCTs in the four studies included in our systematic review were reported to be effective with significant outcomes, particularly in the treatment groups [35,36,38,39]. The findings in similar studies conducted previously also confirmed the efficacy of rehabilitation treatment through RCTs [44,57,58]. However, RCTs may not be feasible for complex rehabilitation programs under certain circumstances of large-scale earthquakes with large populations affected. It is important that non-RCT designs other than experimental, retrospective and cohort approaches identified in our systematic review be carefully considered while selecting the different rehabilitation interventions for earthquake-affected survivors, for achieving optimal and better outcomes. Of note, the successful implementation of interventions—whether based on RCT or non-RCT—requires intersectoral collaboration among different service providers, and where possible, designating some responsibilities [22,60]. In this regard, although most of the HRR interventions identified in the 11 studies were championed by academic-based authors, they were coordinated in one way or another with other stakeholders such as the Chinese government, charities, foundations, hospital staff and other volunteers—whose roles mainly related to the funding and data collection. This notwithstanding, our systematic review found that there was no consensus among stakeholders in the 11 studies on the specific interventions or even the unified tools for assessing the HRR needs of survivors. A similar case was echoed in a systematic review by Hong and Efferth (2016) [15], where no particular tool for assessing PTSD among 2008 Wenchuan earthquake survivors was agreed upon by the different studies included in it.

### Study Limitations

Generally speaking, HRR in post-earthquake scenarios and other disasters can be diverse. Thus, some eligible and relevant studies may have been excluded, as a result narrowing the scope and the findings of our systematic review, given that the study time was restricted to between 2008 and 2018, only five English databases were searched, and only papers published in English were considered. However, excluding the papers published in languages (and mainly Chinese) other than English was inevitable, as much as our initial intention was to search and review both English and Chinese studies, since the latter consisted of various inconsistencies and particularly poor methodological designs. Additionally, as noted above, our study was retrospective and focused on the 10th anniversary of the 2008 Wenchuan earthquake, and as such, some recent studies published beyond 2018 may have been left out. Aside from these limitations, our systematic review provides an overview of some of the HRR interventions that have been rendered to the survivors of the 2008 Wenchuan earthquake.

## 5. Conclusions

Based on the 11 studies included in our systematic review, different HRR interventions for the survivors or victims of the 2008 Wenchuan earthquake, although being predominated by physical rehabilitation, were reported to be effective. However, it is inconclusive as to which specific intervention(s) and tool(s) was/were much more effective than others for rehabilitating the survivors of the earthquake. One explanation for this is the heterogeneity of the 11 studies—they are without a consensus, since each had different objectives, participants, outcomes, designs and implementations of the intervention. What ought to be noted instead, particularly in the post-earthquake scenario, is that rendering some specific HRR may be complex, and achieving better outcomes requires tailoring the interventions used based on components. They should not be limited only to different models of rehabilitation treatment, but also the collaboration between different services providers, patients’ needs and consent, existing rehabilitation capacity and other resources—above all, premising them on the existing global rehabilitation standards. Besides, the rehabilitation needs of the vulnerable groups and populations at greater risk must be quickly identified and prioritized. Although over ten years have passed since the occurrence of the 2008 Great Wenchuan earthquake, its devastating impacts continue to reverberate in the minds of survivors, which calls for their continuous rehabilitation care. Therefore, the rehabilitation interventions adopted need to be accurately assessed over a given time to be effective, and this can be achieved by conducting more robust research that employs appropriate methodological designs and augments our systematic review.

## Figures and Tables

**Table 1:** Details of the final studies that were included in the systematic review.

	Author/Year	Study Design	Study Objective(s)/Aim(s)	Intervention(s)	Participant Inclusion and Type of Subject	Subject Gender/Age	Study Outcome Measurement	Recommendations/Primary Findings
1	Zang Yinyin [36]/ 2013	RCT	Evaluate the efficacy of NET as a short-term treatment for PTSD earthquake survivors.	NET	22/PTSD	Male = 5; Female = 17/ E = 56.64 & C = 54.82	PTSD symptoms, general mental health, social support, coping style and posttraumatic change	Effectiveness in treating post-earthquake traumatic symptoms in adult Chinese earthquake survivors
2	Ying Chen [38]/ 2014	RCT	Compared the treatment effectiveness of short-term CBT with a general supportive intervention and with a control group of non-treatment.	CBT	40/Adolescence	Male = 13; Female = 27/Age = 14.50	Psychological resilience, PTSD & depression	CBT was effective in reducing PTSD and depressive symptoms, improved psychological resilience
3	Meng XianZe [35]/ 2012	RCT	Investigated effects of a Chinese herbal formula on GPS in earthquake survivors with PTSD	12g packages of granulated XTJYF or placebo twice a day for eight weeks. Instructed to drink the contents dissolved in warm, boiled water.	268/PTSD	Male = 71; Female = 174/E = 51.2 & C = 51.0	Self-reporting psychological distress	XTJYF may be an effective and safe treatment option for improving GPS in patients with PTSD
4	Jiang Ruifang [39]/ 2014	RCT	Test the efficacy of IPT delivered by trained local personnel compared with TAU for PTSD and MDD among adults affected by the Sichuan 2008 earthquake.	IPT	49/PTSD, MDD	Male = 35; Female = 14/ E = 24.79 & C = 36.05	CAPS & SCID	IPT is a promising treatment for reducing PTSD and depression
5	Xia Zhang [16]/ 2013	Longitudinal quasi-experimental study	Evaluated the effectiveness of the NHV program	NHV Rehabilitation Services Program	510/Disabling injuries	Male = 179; Female = 331/ E = 55.2:L = 53.4 & C = 51.8	BI	NHV improved the long-term physical functioning of Sichuan earthquake survivors with disabling injuries
6	Jun Ni [17]/2013	Cross-sectional survey	Evaluated the effectiveness of a rehabilitation intervention on PDF and PTSD in fractured victims to identify risk factors for PTSD.	Regular rehabilitation	459/Fracture	Male = 16; Female = 296/<30 = 52:30–50 = 129:50–70 = 196: > 70 = 82	PCL-C, Muscle strength, ROM, sensory function and sit-to-stand balance capacity	PDF and PTSD were significantly reduced by the rehabilitation intervention
7	Li Ling [20]/ 2015	Prospective cohort study	Examined the development and determinants of long-term outcomes for earthquake victims with amputations	Institution-based rehabilitation	45/Amputation	Male = 22; Female = 23/Age = 43.5	VAS, BI, SF-36 and Life Satisfaction Questionnaire-11	While amputees’ functioning and pain were improved over time, QoL and life satisfaction did not change
8	Li Wing Sum [40]/ 2018	Cross-sectional study	Identified factors associated with successful functional recovery of bilateral amputees’ age.	Stand Tall rehabilitation programme	17/Traumatic bilateral amputation	Male = 8; Female = 9/ Age = 26.59	Mobility, prosthesis use and health-related QoL	Rehabilitation makes better in adjustment and QoL in bilateral lower limb amputees
9	Li Yongqiang [19]/ 2012	Non-RCT clinical study	Assessed the effect of individualized physical rehabilitation programming on victims’ functional recovery.	Physical rehabilitation programming	51/ SCI	Male = 21; Female = 30/age ranged 11–77 years - with the majority of persons between 18 and 60 years of age.	Ambulation, wheelchair mobility and ADL	Earthquake victims with SCI improved on physical rehabilitation programme
10	Zhu Zhuohong [37]/ 2014	Non-RCT clinical study	Investigated the treatment effects of calligraphy therapy on child survivors of Sichuan earthquakes	Calligraphy training 1 hour a day	210/PTSD	Boys = 105; Girls = 105/children in the fourth and fifth grades	PTSD, calligraphy therapy, salivary cortisol, salivary cortisol levels and arousal scores	Successful calligraphy treatment is an effective and culturally congruent system of intervention
11	Wang Z [41]/2016	Non-RCT clinical study	Examined the use of a Web-based self-help intervention program and investigate the relationship between program use and user characteristics	A Web-based self-help intervention	146/General	Male = 34; Female = 112/Age range:16-25 = 30:26-40 = 64:41-70 = 52	PDS, SCL-D, CSE, CSS & SFI	Both individual (e.g. demographic, health problems, psychological) and social factors (e.g. social functioning, social support) should be considered when delivering Web-based interventions

Notes: CBT: cognitive-behavioral therapy; NET: narrative exposure therapy; XTJYF: Xiao-Tan-Jie-Yu-Fang; GPS: General Psychological Status; IPT: Interpersonal Psychotherapy; TAU: Treatment As Usual; MDD: Major Depressive Disorder; PTSD: Posttraumatic Stress Disorder; CAPS: Clinician Administered PTSD Scale; SCID: Structured Clinical Interview for DSM-IV; NHV:N = non-governmental organizations (NGOs), local health departments (H), and professional rehabilitation volunteers (V); BI: Barthel Index; PDF: Physical Dysfunction; PCL-C: The PTSD Checklist-Civilian Version; ROM: Joint Range of Motion; VAS: Visual Analogue Scale; ABCF: Activity-Based Cognitive Fear Reduction; FSSC: Fear Survey Schedule for Children; PDS: Posttraumatic Diagnostic Scale; SCL-D: Symptom Checklist 90-Depression; CSE: Trauma Coping Self-Efficacy Scale; CSS: Crisis Support Scale; QoL: Quality of Life; and SFI: Social Functioning Impairment.

**Table 2:** Quality assessment of the non-RCT studies based on ROBINS-I.

	Author(s)/Year	Bias Due to Confounding	Bias in Selection of Participants into the Study	Bias in Measurement of Intervention(s)	Bias Due to Departures from Intended Intervention(s)	Bias Due to Missing Data	Bias in Measurement of Outcome(s)	Bias in Selection of Reported Result(s)	Overall Bias
1	Li Yongqiang/ 2012	Low	Moderate	Low	Low	Moderate	Moderate	Low	Moderate
2	Xia Zhang/ 2013	Low	Moderate	Low	Moderate	Moderate	Moderate	Low	Moderate
3	Jun Ni/ 2013	Moderate	Moderate	Low	Moderate	Low	Moderate	Low	Moderate
4	Zhu Zhuohong/ 2014	Low	Low	Moderate	Moderate	Low	Moderate	Low	Moderate
5	Li Ling/ 2015	Serious	Moderate	Serious	No information	Critical	Low	Low	Critical
6	Wang Z/ 2016	Moderate	Moderate	Moderate	No information	Low	Moderate	Low	Moderate
7	Li Wing Sum/ 2019	Moderate	Serious	Moderate	Low	Low	Moderate	Low	Serious
